# An immobilization and localization technique for SRT and IMRT of intracranial tumors

**DOI:** 10.1120/jacmp.v3i4.2556

**Published:** 2002-09-01

**Authors:** Leonid B. Leybovich, Anil Sethi, Nesrin Dogan, Edward Melian, Mathew Krasin, Bahman Emami

**Affiliations:** ^1^ Department of Radiation Oncology, Stritch School of Medicine Loyola University Chicago 2160 South First Ave. Maywood Illinois 60153; ^2^ Edward Hines Jr. VA Hospital Hines Illinois 60141

**Keywords:** immobilization, intensity modulated, stereotactic, 3D CRT, radiotherapy

## Abstract

A noninvasive localization and immobilization technique that facilitates planning and accurate delivery of both intensity modulated radiotherapy (IMRT) and linac based stereotactic radiotherapy (SRT) of intracranial tumors has been developed and clinically tested. Immobilization of a patient was based on a commercially available Gill‐Thomas‐Cossman (GTC) relocatable frame. A stereotactic localization frame (LF) with the attached NOMOS localization device (CT pointer) was used for CT scanning of patients. Thus, CT slices contained fiducial marks for both IMRT and SRT. The patient anatomy and target(s) were contoured on a stand‐alone CT‐based imaging system. CT slices and contours were then transmitted to both IMRT and SRT treatment planning systems (TPSs) for concurrent development of IMRT and SRT plans. The treatment method that more closely approached the treatment goals could be selected. Since all TPSs used the same contour set, the accuracy of competing treatment plans comparison was improved. SRT delivery was done conventionally. For IMRT delivery patients used the SRT patient immobilization system. For the patient setup, the IMRT target box was attached to the SRT LF, replacing the IMRT CT Pointer. A modified and lighter IMRT target box compatible with SRT LF was fabricated. The proposed technique can also be used for planning and delivery of 3D CRT, thus improving its accuracy. Day‐to‐day reproducibility of the patient setup can be evaluated using a SRT Depth Helmet.

PACS number(s): 87.53.Kn, 87.53Ly, 87.56.Da

## INTRODUCTION

Conformal single fraction stereotactic radiosurgery (SRS), multiple fraction stereotactic radiotherapy (SRT), and intensity modulated radiotherapy (IMRT) of intracranial lesions require a high degree of accuracy in the target and normal structures localization. Similar accuracy is required for patient immobilization and positioning during treatment delivery.^1^ ^–^ ^5^

A variety of immobilization and localization devices were reported in the literature. Invasive devices for intracranial tumors such as the Leksell,^6^ Reichert‐Mundinger,^7^ Brown‐Roberts‐Wells (BRW)^8^ frames, and the TALON system^9^ for IMRT can provide positional accuracy of 1 mm. However, except for the TALON system, invasive devices require patients to keep a stereotactic frame affixed to their skull for the entire duration of fractionated radiotherapy.^10^ ^,^ ^11^ The invasive nature of the aforementioned immobilization devices has prevented their wide acceptance for fractionated treatments.^12^

Different types of thermoplastic masks and cradles were investigated for patient immobilization for fractionated conformal radiotherapy. According to the majority of publications, accuracy of patient repositioning using thermoplastic devices varies from at least 2 mm to more than 3 mm.^4^ ^,^ ^12^ ^–^ ^14^

For SRT, relocatable frames with 1 mm accuracy of patient repositioning were developed.^15^ ^,^ ^16^ Based on the Gill‐Thomas‐Cosman (GTC) Relocatable Head Frame,^17^ a noninvasive localization and immobilization technique that provided accuracy of patient repositioning on the order of 1 mm for IMRT and SRT methods was developed and tested in this work. This immobilization technique can also be used for 3D CRT.

Since many radiation oncology clinics are equipped with stand‐alone computed tomography (CT)‐based imaging systems (AcQSim, for instance), contouring of the patient anatomy may be done on such systems. Contoured structures are then transmitted to treatment planning systems (TPSs). In the proposed technique, the CT images contain fiducial marks for all conformal radiotherapy methods. Therefore, the same set of images could be used in 3D CRT, IMRT, and SRT TPSs (in our department, FOCUS, CORVUS, and X‐knife TPSs, respectively) for concurrent development of treatment plans. Because all TPSs used the same structure outlines, a more accurate comparison of competing treatment methods is possible. Thus, a treatment method that produces more favorable target coverage and normal tissues sparing may be selected. With the proposed immobilization technique, the need for modality‐specific imaging of a patient and fabrication of immobilization device was eliminated.

## METHODS AND MATERIALS

The most precise localization and immobilization technique implemented in the Nomos‐Peacock IMRT unit is based on an invasive “NOMOS TALON” system. The TALON is mounted on the patient skull using two self‐tapping titanium screws.^9^ These screws stay in the skull for several weeks. For patient immobilization, the TALON body is attached to the NOMOS adjustable bracket (NOMOGrip), which can be mounted on either the CT, or the treatment couches. Thus, the TALON system allows accurate repositioning of the patient.^9^ The NOMOS coordinate system is defined by the CT marker (CT pointer), which is attached to the NOMOGrip opposite to the TALON during CT scanning. The CT marker could be also used to define the coordinate system for 3D CRT. For patient positioning during treatment, the target box replaces the CT pointer. The initial position of the patient (and the treatment couch) is set according to the lines on the target box. Then the treatment couch is advanced according to the treatment plan for sequential delivery of treatment arcs (tomotherapy or TIMRT) or for delivery of static step‐and‐shoot IMRT (SIMRT) or 3D CRT.

One of the relocatable frames developed for SRT (GTC Relocatable Head Frame) uses the dental impression of a patient's upper teeth (dental appliance), a headrest with an individualized occipital pad, and adjustable straps.^17^ For the CT scan, the GTC relocatable frame is rigidly attached to the CT scanner couch and the BRW Localizer Frame (BRW‐LF) is clamped to the GTC frame. The BRW coordinate system is specified by images of nine localization rods on CT slices.

To immobilize the patient for treatment delivery, the GTC frame is fixed to the Linac Couch Mount Assembly (LCMA) and, for the patient positioning, the Linac Target Locator Frame (LTLF) is attached to the GTC frame. The set‐up lines on the LTLF should be aligned with the treatment room lasers.

**Figure 1:**
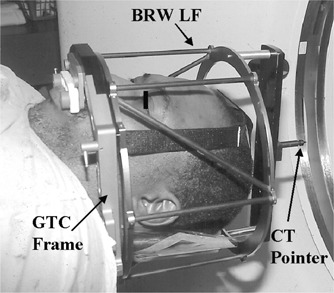
CT scanning of a patient. The GTC relocatable frame contains fiducial marks for both SRT and IMRT.

**Figure 2:**
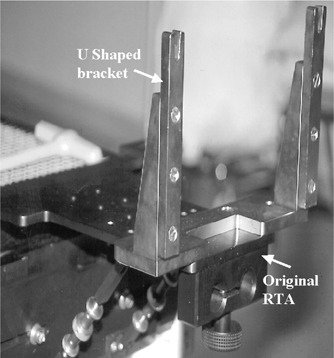
Modification of the radiation couch extension (RTA) in order to accommodate the proposed immobilization technique for IMRT (and 3D‐CRT) delivery.

To combine both coordinate systems, the NOMOS CT pointer was attached to the top of the BRW‐LF (Fig. 1). The only difference in the scanning protocol for SRT was that, in addition to BRW‐LF, the CT scan had to include the CT pointer. The CT images were then transported to the imaging system (AcQSim) where contouring of the patient was done. Contoured CT slices were transmitted (using a DICOM‐3 interface) to treatment planning computers (CORVUS and X‐Knife and, if necessary, to FOCUS). The images were further processed according to the software requirements of each system. The patient setup for IMRT (and 3D CRT) delivery required several simple modifications of the regular NOMOS procedures. For patient immobilization, an SRT *U*‐shaped bracket was attached to the radiation couch extension [NOMOS radiation table adapter (RTA)], see Fig. 2.

**Figure 3:**
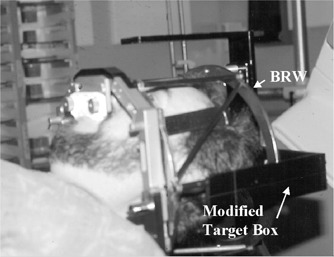
Patient setup for IMRT delivery.

This allowed clamping of the GTC frame to the RTA. For patient setup, the BRW‐LF was attached to the GTC frame and a new, modified target box was attached to the BRW‐LF frame (Fig. 3). A modified, lighter target box was fabricated because the BRW‐LF did not fit into the original target box and, perhaps, was too heavy for the BRW‐LF.

To check the correspondence between reconstructed structure volumes, treatment plans were run for 16 patients with brain tumors on SRT and IMRT TPSs and volumes of different structures as represented by TPSs were inter‐compared. Structure shapes varied from approximately spherical (eyes), or cylindrical (brain stem) to highly irregularly shaped optic chiasms and targets. Structure volumes varied from ~0.6cc (optic chiasm and optic nerves) to ~20cc (brain stem and targets).

The TPSs used (i) the same set of contours (obtained from a stand‐alone imaging system) and (ii) contours delineated on each TPS separately by the same person. The accuracy of patient repositioning was determined by Depth‐Helmet^18^ measurements. A computer program (written in fortran) converted Depth‐Helmet measurements into displacements in a Cartesian coordinate system.

## RESULTS

**Figure 4:**
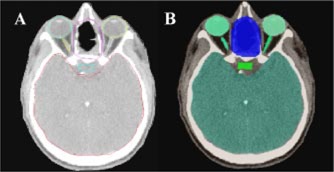
(Color) Brain structures and the target representation in X‐knife TPS (left) and Corvus TPS (right). The same contour set was used in both TPSs.

Figure 4 shows patient anatomy contoured on the AcQSim system and further processed on X‐Knife TPS (left) and IMRT TPS (right). The contours look quite similar in shapes and areas occupied by them. However, there were small (1.2% on the average) differences between structure volumes calculated by CORVUS and X‐knife, respectively. This difference may be attributed to different volume calculation algorithms (contour‐based on X‐Knife versus voxel‐based on CORVUS). The difference between volumes calculated for the same object increased to 3% (on the average) when organs were delineated on each TPS separately. However, for very small structures (optic nerves and optic chiasm) this difference was ~5%.

Most patients with brain tumors were treated with SRT. However, some (10–15%) brain tumor patients were treated with IMRT due to more conformal, as compared to SRT, dose distribution. Accuracy of the patient repositioning, treated with either SRT or IMRT was calculated, based on the Depth‐Helmet measurements, to be 1±0.3mm.

## DISCUSSION AND CONCLUSION

CORVUS IMRT Treatment Planning Software can accept several types of localization and immobilization devices (Radionics is not among them), but only the TALON system provides localization and immobilization accuracy on the order of 1 mm. However, the invasive character of the TALON system prevents its wide acceptance by the radiation oncology community.

The noninvasive GTC relocatable frame used in SRT may provide the accuracy of patient repositioning on the order of 1 mm. The proposed system for patient localization and immobilization uses all the advantages of the SRT immobilization and localization methods (1 mm accuracy, noninvasiveness, day‐to‐day patient position verification with the Depth Helmet). Both SRT and IMRT can be used for treating brain tumors located in close proximity to brain critical structures. In addition, this system may be also used with 3D CRT, thereby improving accuracy of dose delivery to brain tumors. Since this method provides coordinate systems for all aforementioned treatment techniques, no additional treatment‐specific imaging of a patient and fabrication of immobilization device is necessary. If all TPSs use the same contour set, the proposed method may allow a more accurate comparison of the treatment plans produced for different treatment methods.

This immobilization system can be also used for single‐fraction treatments. However, the GTC relocatable frame requires fabrication of a patient‐specific dental appliance and occipital pad. To verify reproducibility of the frame position relative to the patient head, Depth‐Helmet measurements should be taken over the course of several days prior to any treatment‐related procedures. Frequently, some adjustments in dental appliance or occipital pad positions are necessary. Thus, customization of the relocatable frame is a long, laborious process and therefore this frame is used primarily for multifraction treatments. For a single‐fraction treatment, invasive devices are considered to be acceptable because their placement takes 30 min or less and a patient undergoes this procedure only once.
